# A community mobilisation intervention to prevent violence against women and reduce HIV/AIDS risk in Kampala, Uganda (the SASA! Study): study protocol for a cluster randomised controlled trial

**DOI:** 10.1186/1745-6215-13-96

**Published:** 2012-06-29

**Authors:** Tanya Abramsky, Karen Devries, Ligia Kiss, Leilani Francisco, Janet Nakuti, Tina Musuya, Nambusi Kyegombe, Elizabeth Starmann, Dan Kaye, Lori Michau, Charlotte Watts

**Affiliations:** 1 Gender Violence and Health Centre, London School of Hygiene and Tropical Medicine, 15-17 Tavistock Place, London, WC1H 9SH, UK; 2 Booz Allen Hamilton, 901 15th Street NW, Washington, DC, 20005, USA; 3 Raising Voices, Plot 16 Tufnell Drive, Kamwokya, PO Box 6770, Kampala, Uganda; 4 Centre for Domestic Violence Prevention, Plot 16 Tufnell Drive, Kamwokya, PO Box 6770, Kampala, Uganda; 5 Department of Obstetrics and Gynaecology, School of Medicine, Makerere University College of Health Sciences, P.O. Box 7072, Kampala, Uganda

**Keywords:** Violence against women, Intimate partner violence, Domestic violence, HIV/AIDS, SASA, Community mobilisation, Behaviour change, Gender, Community randomised trial, Uganda

## Abstract

**Background:**

Gender based violence, including violence by an intimate partner, is a major global human rights and public health problem, with important connections with HIV risk. Indeed, the elimination of sexual and gender based violence is a core pillar of HIV prevention for UNAIDS. Integrated strategies to address the gender norms, relations and inequities that underlie both violence against women and HIV/AIDS are needed. However there is limited evidence about the potential impact of different intervention models. This protocol describes the SASA! Study: an evaluation of a community mobilisation intervention to prevent violence against women and reduce HIV/AIDS risk in Kampala, Uganda.

**Methods/Design:**

The SASA! Study is a pair-matched cluster randomised controlled trial being conducted in eight communities in Kampala. It is designed to assess the community-level impact of the SASA! intervention on the following six primary outcomes: attitudes towards the *acceptability of violence against women* and the *acceptability of a woman refusing sex* (among male and female community members)*;* past year *experience of physical intimate partner violence* and *sexual intimate partner violence* (among females); *community responses to women experiencing violence* (among women reporting past year physical/sexual partner violence); and past year *concurrency of sexual partners* (among males). 1583 women and men (aged 18–49 years) were surveyed in intervention and control communities prior to intervention implementation in 2007/8. A follow-up cross-sectional survey of community members will take place in 2012. The primary analysis will be an adjusted cluster-level intention to treat analysis, comparing outcomes in intervention and control communities at follow-up. Complementary monitoring and evaluation and qualitative research will be used to explore and describe the process of intervention implementation and the pathways through which change is achieved.

**Discussion:**

This is one of few cluster randomised trials globally to assess the impact of a gender-focused community mobilisation intervention. The multi-disciplinary research approach will enable us to address questions of intervention impact and mechanisms of action, as well as its feasibility, acceptability and transferability to other contexts. The results will be of importance to researchers, policy makers and those working on the front line to prevent violence against women and HIV.

**Trial registration:**

ClinicalTrials.Gov NCT00790959

## Background

Intimate partner violence (IPV) against women is widely recognised as a major global public health problem, with the World Health Organisation (WHO) multi-country study on women’s health and domestic violence highlighting the scale and extent of the problem in 10 countries around the world [1]. The gendered nature of the HIV/AIDS epidemic has also received increased attention in recent years [2], with women now constituting 60% of adults living with HIV in sub-Saharan Africa [3]. Epidemiological evidence points to important links between these two epidemics. In particular, recent analyses of cohort data looking at incident HIV infection [4,5] have found IPV to be an independent risk factor for HIV.

There are multiple posited mechanisms through which the IPV/HIV association occurs. Coerced sex and other forms of sexual violence may contribute directly to a woman’s HIV risk, being a potential route of exposure to the virus as well as increasing the chances of HIV transmission if lacerations or other genital trauma occur [6]. Women in violent relationships are also less likely to be able negotiate the frequency or circumstances of sex, thus exacerbating their HIV risk. Evidence points to a number of indirect mechanisms of association between IPV and HIV, and also potential shared risk factors. Women in violent relationships may be more likely to have multiple partners [7,8], have unprotected sex [9], and have had an early sexual debut. Substance use is also higher among female survivors of IPV [8,10,11]. Similarly, research suggests that men who are violent towards their partner are more likely to have multiple partners [12], visit sex workers [13], refuse to use condoms [12,14], and to have a sexually transmitted infection (STI) [15]. High alcohol intake is also an independent risk factor for both intimate partner violence and HIV [16]. These factors increase a man’s own risk of HIV and thereby influence the subsequent risk that he will transmit infection to his female partner (and victim of IPV).

Underpinning many of these mechanisms, are inequitable gender roles and relations. Economic and social gender inequities, together with norms and expectations about how women and men should behave within and outside intimate relationships, influence risk of IPV and HIV in a number of ways. Notions of masculinity prevalent in much of the world condone or attach status to men who exhibit dominance over women, engage in sexual conquests and take risks. It is therefore not surprising that men who endorse more traditional views about masculine roles and behaviours have been shown to be more likely than other men to perpetrate IPV and to engage in higher risk sexual behaviours [14,17] - both behaviours being manifestations of the same model of masculinity. Similarly, female gender roles, which assume women’s subservience to men and power imbalances in sexual relations, often sanction the acceptability of violence against women and can increase vulnerability to HIV [18] - for example if a woman’s power to negotiate condom use is limited through fear of implying promiscuity, transgressing gender norms and incurring violent repercussions [9,19]. Research has shown that women who believe that there are circumstances where a man can be violent towards their partner are more likely to experience IPV [20], and there is also evidence that women in less equitable relationships are at increased risk of HIV. For example, Jewkes *et al*. (2010) found that inequity of power within a relationship was an independent risk factor for incident HIV infection among women, even after controlling for partnership duration and other indicators of risk behaviour [4].

Against a backdrop of gender inequity, an HIV diagnosis and/or its disclosure may also put a woman at increased risk of IPV [6]. In turn, fears of violent repercussions may prevent women from taking up HIV testing or disclosing their HIV status [21-23]. The El-Bassel *et al*. (2005) cohort study among methadone-maintained women in New York City found that self-reported STIs in the previous six months were associated with a woman’s subsequent risk of IPV, an association hypothesized to be attributable to relationship conflict caused by the disclosure of an STI and its potential association with infidelity [11].

The need for HIV prevention efforts that more explicitly incorporate programme elements to address gender inequality and violence, has been repeatedly articulated, and the elimination of sexual and gender-based violence has been identified by the Joint United Nations Programme on HIV/AIDS (UNAIDS) as being one of the core pillars of HIV prevention [24]. In the field of both IPV and HIV, the importance of addressing issues of gender roles and inequality has often been stressed. Despite this rhetoric however, responses to HIV and IPV often remain separate, and there has been relatively limited investment in prevention strategies that seek to tackle their shared, more upstream determinants.

### Gender focused interventions - the evidence to date

In recent years, only a handful of violence and HIV prevention interventions that seek to challenge prevailing gender norms and/or directly empower women have been subject to rigorous impact evaluation.

The Intervention With Microfinance for AIDS and Gender Equity (IMAGE) Study, set in the rural South African province of Limpopo, assessed the impact of an intervention that combined a microfinance programme for poor women with participatory gender and HIV training. Using a cluster randomised design involving four intervention and four control villages, the study found that over two years the intervention led to a 55% reduction in past year IPV among intervention participants (adjusted risk ratio 0.45, 95% confidence interval (CI) 0.23 to 0.91) [25]. Among younger intervention recipients, the intervention was also associated with statistically significant increases in uptake of HIV testing and HIV related communication in the household, and a reduction in prevalence of unprotected sex at last intercourse with a non-spousal partner [26]. Positive intervention impacts were also observed on a range of indicators of female empowerment, including increased autonomy in decision making, increased financial confidence, and increased participation in collective action, indicators which were secondary outcomes of the trial and conceived as potential pathways through which the intervention may lead to reduced violence [27]. Based on the hypothesis that intervention effects would diffuse beyond direct loan recipients, the trial also assessed behaviours and HIV incidence in the broader community. Over the three-year study no impact on HIV incidence among the wider community was observed. This was not surprising given the relatively short follow-up and thus limited chance for the diffusion of intervention effects. The intervention also achieved widespread mobilisation around HIV and violence against women, and alongside the large scale replication of the microfinance programme, IMAGE has now been scaled up to 15,000 households.

Stepping Stones, a participatory HIV prevention programme which aims to improve sexual health through building more gender-equitable relationships, was also evaluated using a cluster randomised trial involving 35 intervention clusters and 35 control clusters. The study was implemented in the Eastern Cape province of South Africa, with the aim of reducing HIV and Herpes simplex virus type 2 (HSV-2) incidence, and the prevalence of sexual risk behaviours and IPV. After a two year follow-up period, the intervention was not associated with a reduction in HIV incidence, but it was associated with a 33% reduction in HSV-2 incidence, with a similar effect observed for men and women (adjusted incidence rate ratio 0.67, 95% CI 0.46 to 0.97). Among male respondents, reductions were also seen in sexual risk behaviours and in reported levels of perpetration of IPV against women [28].

In Rio de Janeiro, Brazil, a quasi-experimental study undertaken by the Horizons Program and Instituto Promundo assessed the impact of a participatory intervention to address traditional norms of masculinity and the acceptability of violence among young men (aged 14 to 25 years). Three cohorts, the first receiving interactive group education sessions led by adult male facilitators, the second receiving these group education sessions alongside a community-wide social marketing campaign to promote condom use, and the third being controls (receiving the intervention after a 6-month delay), were surveyed prior to intervention activities, and 6 and 12 months later. After one year, the two intervention cohorts both saw a significant reduction in reported inequitable gender norms, a decrease in reported STI symptoms, and an increase in reported condom use at last sex with a primary partner [29].

Another ongoing intervention study is a cluster randomised trial of an IPV primary prevention intervention in the Cote d’Ivoire: Men and Women in Partnership. The intervention comprises men’s discussion groups which aim to prevent violence against women through: increasing men’s knowledge about the impact of violence; shifting beliefs and behaviour within the relationship and household in a more gender equitable direction; developing anger management techniques; and increasing women’s disclosure of IPV. The end-line survey is scheduled for February 2012.

### Evaluating complex community-focused interventions

The scarcity of evidence around gender-focused interventions derives in part from the difficulties inherent in designing interventions to tackle deeply entrenched structural factors, such as gender norms and inequality. It also reflects the challenges involved in the evaluation of complex community-focused interventions that have the aim of promoting widespread social change.

Increasingly, policy makers and donors are demanding evidence on the effectiveness of complex public health interventions that is of an equivalent rigor to the evidence base in biomedical fields. While randomised controlled trials have long been the gold standard method of evaluation for biomedical interventions, community randomised trials are increasingly advocated for the evaluation of community focused interventions. Cluster randomised trials (CRTs) have been used to assess the impact of several HIV and IPV interventions in recent years [25,28,30-33], including for example, the impact of strengthened STI treatment services on HIV incidence in Tanzania, and the impact of youth-focused HIV prevention activities [34,35].

However, the implementation of such trials is fraught with challenges. Resource constraints and programmatic limitations may limit the number of communities that can be included in the study; existing service structures, political interests and ethical objections may pose impediments to randomisation; the length of time needed for certain types of change to occur, especially the types of change which are often the focus of structural interventions, may be so long as to make intervention impact on relevant outcomes impossible to measure within the limited time-frame of a study [36]. Furthermore, because the nature of these interventions and their impacts are often so dependent on social context, it may not be easy to assess the degree to which study results can be generalized to other settings [37] in the absence of additional data on intervention process and mechanisms of causality. Thus, while there is growing consensus that a CRT should be the method of choice where possible, there is also increasing recognition of the need for trials to include extensive process and qualitative data collection to complement quantitative approaches. These additional elements allow researchers to better understand the processes through which the intervention is delivered and the mechanisms by which change occurs. This in turn also helps inform how the intervention and its impacts may transfer to other settings.

Further intervention research is needed, both to assess the impacts of different models of IPV and HIV prevention interventions, and to contribute to the development of methodological approaches to evaluate upstream social interventions.

### The SASA! study

This protocol describes the SASA! study, an evaluation of the SASA! intervention to prevent violence against women and reduce HIV/AIDS risk. It is one of relatively few CRTs to evaluate a gender focused intervention attempting to change underlying social norms that support both violence against women and HIV/AIDS. The study is set in Kampala, Uganda, a setting with a high prevalence of IPV and HIV/AIDS. HIV prevalence among women attending antenatal clinics stands at just over 6% in Uganda [38], and studies suggest that incidence may again be on the rise [39,40]. Prevalence is higher among women than men, and higher in urban than rural areas [41]. Uganda is also affected by high levels of IPV; in the 2006 Demographic and Health Survey (DHS) 59% of ever-married women aged 15 to 49 years reported having experienced physical and/or sexual violence by an intimate partner at some point in their lives [42].

## Methods/Design

### The SASA! intervention

**Figure 1:**
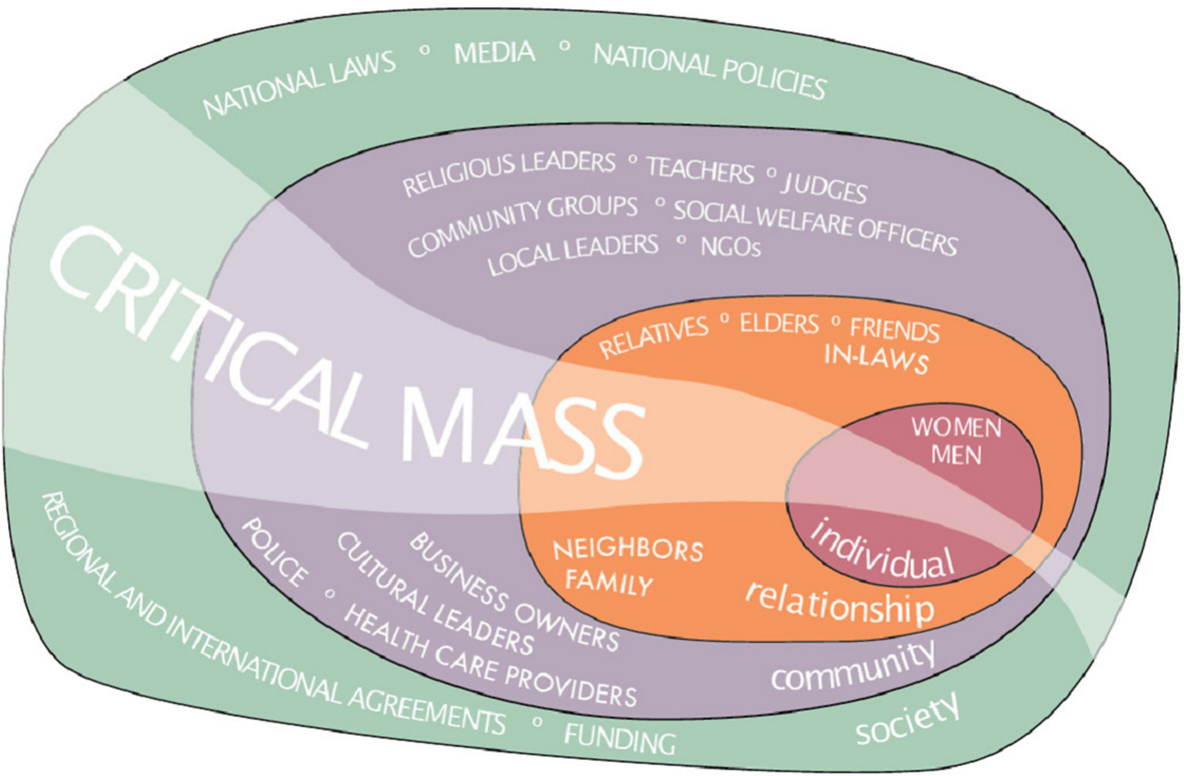
Ecological model as applied to IPV prevention.

SASA! - *An Activist Kit for Preventing Violence against Women and HIV *[43], was designed by Raising Voices and is being piloted and implemented by the Centre for Domestic Violence Prevention (CEDOVIP) in Kampala. The intervention uses a community mobilisation approach to try to change the community attitudes, norms and behaviours that underlie power imbalances between men and women and support both HIV risk behaviours and the perpetration of violence against women. The intervention takes a holistic approach, that explicitly recognises that IPV is the result of a complex interplay of factors operating at the individual, relationship, community and societal levels [44] - therefore interventions to prevent it must engage with and achieve change at each of these levels (see Figure 1).

SASA! also draws heavily upon a social-level adaptation of the Stages of Change Theory [45], explicitly taking communities through a four-phase process of change. Indeed, the name SASA! is an acronym for this four phase process:

**Start** – Start thinking about violence against women and HIV/AIDS as interconnected issues and foster power within yourself to address these issues.

**Awareness** – Raise awareness about communities’ acceptance of men’s use of power over women, which fuels HIV/AIDS and violence against women.

**Support** – Support women and men directly affected by or involved in these issues to change.

**Action** – Take action to prevent HIV/AIDS and violence against women.

**Figure 2:**
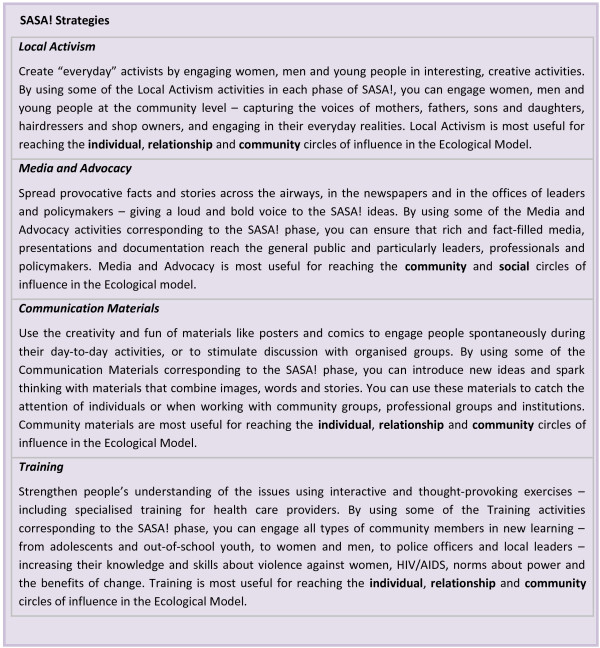
SASA! strategies.

The intervention supports communities through these four phases of change by ensuring that community members are exposed to regular and ongoing mutually reinforcing messages from a variety of formal and informal sources (Figure 2). The focus of the messaging and activities changes as the intervention moves through the different implementation phases. To support this process the SASA! intervention staff work with four groups of actors: community activists (CAs) selected from the more progressive men and women rooted in the community, who work voluntarily to facilitate and promote SASA! activities; community leaders including *ssengas* (traditional marriage counselors) who, as religious, cultural, governmental and other types of local leaders, are encouraged to integrate ideas of gender and power into their leadership roles; professionals such as health care providers and police officers, who provide direct prevention and response services; and institutional leaders who have the power to implement policy changes within their institutions. SASA! entails the selection, training, and mentoring of these individuals and groups, to help improve their knowledge, communication skills, and motivation to participate in mobilising their communities to address gender inequality and violence. As part of this, CAs and leaders are supported by CEDOVIP staff to conduct a range of local activism activities, including public events such as community dramas and discussions, small group activities, and one-on-one ‘quick chats’. Similarly, the police, health workers and other professionals receive training and are supported in efforts to improve the provision of services.

This model of implementation helps ground the intervention in the community, and ensures that it reflects and responds to the reality and everyday lives of community members, as well as enhancing the potential for longer term change.

**Figure 3:**
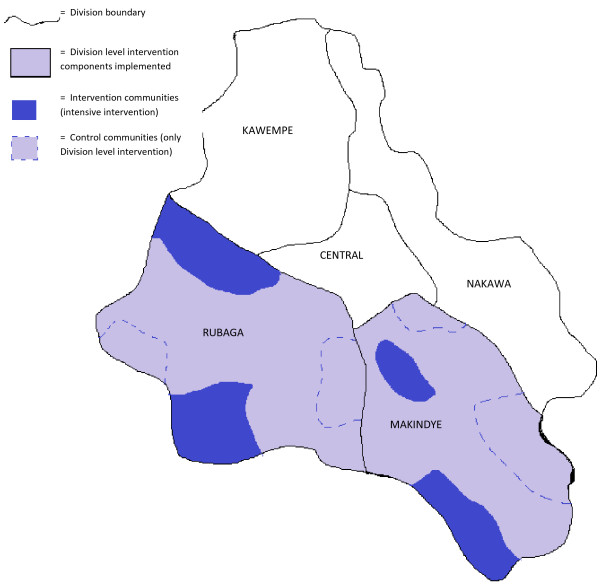
Diagram of Kampala’s administrative Divisions, showing intervention and control sites within the two study Divisions.

Some components of the SASA! intervention (those involving healthcare providers, police, division leadership and, to a limited extent religious leaders) are implemented at the Division level, in accordance with how those services themselves are organised and distributed within Kampala’s administrative structure. The more intensive and comprehensive spectrum of SASA! activities involving the CAs, *ssengas*, cultural leaders and local government leaders occurs at a more local level, within specific parishes. The SASA! study will assess the impact of the full intervention, including the CA- and other parish-level components, as compared to the low-level intervention involving just the Division level components of SASA! (Figure 3).

In the context of this CRT, eight CAs (four male and four female) were recruited in each intervention parish, and trained and supported to deliver the intervention. For sampling purposes (see below for detail) an identical recruitment process was used in control sites, but selected individuals (passive volunteers) went on to receive just one session of basic health education or children’s rights training every three months.

### Theoretical framework and aims

**Figure 4:**
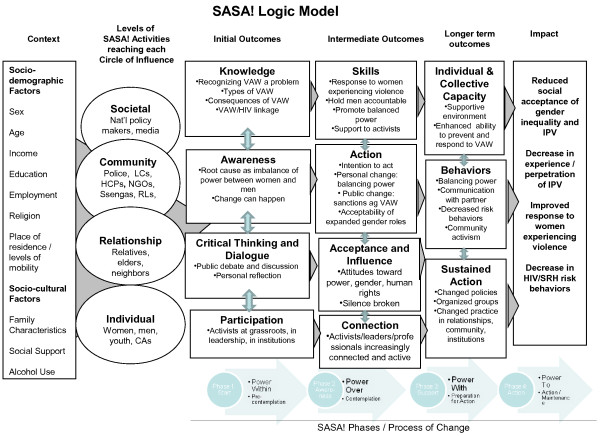
SASA! Logic model.

A logic model for the SASA! intervention is presented in Figure 4. This framework maps out the key contextual variables that may influence intervention impact; the levels of SASA! activities that will be conducted in different spheres of influence; the expected initial, intermediate and longer term outcomes of the intervention; and the long-term sustained impact the intervention is designed to have on the community.

Contextual variables include both socio-demographic variables and factors such as levels of problematic alcohol use - factors that may be associated with both the occurrence of IPV- and HIV-related risk behaviours, but are not in themselves a focus of the SASA! intervention. For example, place of residence and levels of mobility may influence the degree to which community members are exposed to SASA! - in communities where many people live in gated communities, for instance, it may be more difficult for SASA! to achieve substantial coverage. Family characteristics (such as the extent of female headed families) and the existence of social support structures (such as women’s or church groups) that women can potentially draw upon if they experience violence may also be key contextual variables that could influence the impact of SASA!.

As described above, the SASA! intervention model involves working with a broad range of stake holders and community members - individual women and men in the community, people in potential positions of influence who may have a role in helping to prevent or respond to violence (such as neighbours, elders and relatives), as well as people in positions of authority with the potential to provide support to women or men who turn to them for help.

The SASA! model aims to support a process of change, with community-level phases analogous to the processes set out in the individual-level behavior Stages of Change Theory of Prochaska *et al*. (1992) [46]. During the first phase of SASA!, *Start*, the SASA! team (including CAs) starts to foster power within the team to address violence against women and its connections with HIV/AIDS. During this phase, the team gains improved knowledge and awareness, and engages in critical thinking and discussion about: what constitutes violence; the causes and consequences of violence; the underlying links between violence, gender inequality and the misuse of power, and the implications of violence for individuals, families and communities. Gender inequality and social norms about sexual behaviour for men and women are also discussed and opened up to analysis. During this phase, the team engages with only a select few additional community members, but is motivated and empowered to start a positive process of change, to demonstrate the benefits of change to others and facilitate community-wide support for change. Time is also spent understanding the community’s perceptions of violence against women, gender and HIV and building relationships with leaders and gatekeepers who will support and enable the community mobilisation in the subsequent phases.

During the second phase, *Awareness*, the team engages the community to become aware of men’s power over women, and the ways in which this power imbalance (manifested at both the relationship and societal level) perpetuates violence against women and HIV/AIDS risk. This and subsequent phases involve the implementation of a wide range of one-on-one and group-based activities that seek to achieve widespread community participation. The intention of the awareness phase is to spark critical thinking among community members to question the legitimacy of violence against women and gender inequality.

The third phase, *Support*, involves the SASA! team engaging with the community to promote and facilitate individuals joining their *power* with others to confront the dual pandemic of violence against women and HIV/AIDS. This involves fostering supportive networks in which people feel able to seek help and support from others in the community, and where community members work together to support those in need, those trying to change and those speaking out. Community members are supported to feel that they themselves, along with others in their community, can take actions to address gender inequality and violence. Activities focus upon helping people to develop appropriate skills to reduce inequities in their relationships, and to challenge and respond appropriately to violence in their communities. These activities seek to encourage recognition of the ways in which different individuals can address the misuse of power, gender inequality and violence, and the strength that can be generated when they join together with a common aim - as part of this, CAs, leaders and professionals are supported to work more closely together to address violence.

During the final phase, *Action*, the team engages the community in using their power to take action, with the aim of normalising shared power and non-violence, demonstrating its benefits, and as a result, preventing violence against women and reducing HIV/AIDS risk. It is the phase during which the process of change is consolidated, individual and collective action to address violence strengthened, and change institutionalised within local leadership and normative structures.

The comprehensive SASA! intervention is expected to have multiple community-level impacts at the end of these four phases. These are presented in the last column of the logic model: reduced social acceptance of gender inequality and IPV; decreased experience/perpetration of IPV; improved response to women experiencing violence; decreased sexual risk behaviours associated with HIV.

These hypothesised long-term impacts have formed the basis for our selection of primary outcomes in the SASA! study. Within these four areas of impact, six outcomes have been chosen, reflecting the broad scope and complex nature of the intervention as follows:

Reduced social acceptance of gender inequality and IPV

Acceptability of intimate partner violence (among all women; all men)

Acceptability that a woman can refuse to have sex (among all women; all men)

Decrease in experience/perpetration of IPV

Past year experience of physical violence from a partner (among women who have had an intimate partner in the past year)

Past year experience of sexual violence from a partner (among women who have had an intimate partner in the past year)

Improved response to women experiencing violence

Appropriate community response to women experiencing physical and/or sexual IPV in past year (among women who experienced physical and/or sexual IPV in past year)

Decrease in sexual risk behaviours

Past year concurrent sexual partners (among non-polygamous partnered men)

As depicted in the logic model, we hypothesise that changes in gender inequitable norms and norms relating to the acceptability of violence against women will occur prior to, and indeed be pre-requisites for, reductions in IPV and sexual risk behaviours. Change in normative attitudes is also hypothesised to precede improved community responses to women and men affected by violence, which in turn provides another pathway through which longer term reductions in IPV may occur. Shifts in community-wide attitudes and responses to violence and gender inequity are not only conceived as important pathways through which IPV and sexual risk behaviours are reduced, but are also key outcomes in their own right, essential for sustaining behavioural change in the longer term. Currently, norms about the acceptability of IPV and men’s right to have sex within marriage are deeply entrenched - shifts towards more progressive norms around these issues would be a significant intervention success and also attest to the intervention’s potential to support long term and sustained behavioural change within communities.

Reductions in IPV and changes in levels of concurrency are conceived as the outcomes most distal to the intervention. Over the evaluation time-frame, we recognize that achieving a statistically significant reduction in physical IPV is ambitious, but it is nevertheless something that we hope to observe. Sexual IPV is likely to be even harder to impact upon over this short time frame, since social norms supporting sexual entitlement within marriage are deeply entrenched and the challenge thus greater to shift community perceptions towards recognition of sexual coercion as a form of violence – nevertheless sexual IPV is an important outcome to measure.

Concurrency of partners is also likely to take a long time to shift due to deeply entrenched cultural norms around what constitutes acceptable male sexual behaviour. Nevertheless it is an important outcome to include in terms of the intervention’s potential to reduce HIV risk in the longer term. HIV incidence is not chosen as an outcome in this study as reductions in incidence are considered highly unlikely during the relatively short follow-up period of the study. In addition to this, we would have extremely limited statistical power to detect intervention effects on HIV incidence.

### Study objectives

A primary objective of the SASA! study was to assess the impact of the intervention on the following outcomes: acceptability of intimate partner violence (among all women; all men); acceptability that a woman can refuse to have sex (among all women; all men); past year experience of physical violence from a partner (among women who have had an intimate partner in the past year); past year experience of sexual violence from a partner (among women who have had an intimate partner in the past year); appropriate community response to women experiencing physical and/or sexual IPV in past year (among women who experienced physical and/or sexual IPV in past year); past year concurrent sexual partners (among non-polygamous partnered men).

More broadly, the study will also document the scale and nature of programme implementation over time, investigate the processes and causal pathways through which gender-based violence and HIV behaviours are promulgated and change over time, conduct a costing of the SASA! intervention, and explore the cost-effectiveness of SASA!.

This protocol describes in detail how the first study objective will be achieved. The broader study objectives will be addressed in subsequent publications.

### The SASA! study setting

The SASA! Study involves eight sites in two administrative Divisions of Kampala (Makindye and Rubaga), that have not been previously exposed to SASA! intervention activities. Taken together, they encompass approximately 66,500 households with 251,500 inhabitants. The communities selected to participate in the study are located in impoverished areas of Kampala, home to concentrations of people who have migrated to the city from various other parts of the country in search of employment. Baseline data from this study showed that the communities are relatively young compared to the national average, with over 40% of respondents aged 25 years or under [47] - a reflection of the migration of this age-group for work and study opportunities. Rubaga Division accommodates the seat of the Buganda Kingdom, and the dominant tribe in the study communities is Buganda, with the primary language being Luganda. However, Kampala is culturally diverse and communities are home to people of many different tribes and mother tongues, all able to coexist. Catholicism is the most prominent religion, but Islam, Protestantism and Born Again Christianity are also widely followed [47]. Makindye Division is home to the biggest police barracks in Uganda and a police training school.

More than three-quarters of the respondents in our baseline survey lived in rented accommodation, most used a public tap as their main source of drinking water (61%), and fewer than 10% of households had a flush toilet. A high proportion of men (two-fifths) and women (three-fifths) had either not completed or not progressed beyond primary education. Despite living on extremely low incomes, people are resourceful and resilient. In many areas, local leadership is strong and responsive to community concerns.

The study communities are affected by a high prevalence of HIV. The 2004–2005 Uganda HIV/AIDS Sero-Behavioural Survey estimated the prevalence of HIV in Kampala to be 12% among women and 5% among men, higher than the national average [41]. An important determinant of HIV vulnerability is sexual concurrency - where someone with a primary partner also has other extra-marital sexual relationships. Uganda’s early success in addressing the HIV epidemic was in part attributed to locally generated HIV prevention messages that promoted ‘zero grazing’ [48]. However, in tandem with reports that HIV incidence may again be on the rise, the 2004–2005 Uganda HIV/AIDS Sero-Behavioural Survey documented a rise in the proportion of sexually active men reporting multiple partners, from 25% in 2000 to 2001 to 29% in 2004 to 2005. The respective 2004 to 2005 figure for Kampala was 35% [41].

In the SASA! study baseline survey, 27% of women reported having experienced physical and/or sexual violence by an intimate partner in the past year [47]. Traditional norms about male authority remain an important aspect of family life in urban Uganda, even though chronic poverty often prevents men from living up to their ideal roles as providers, and women from dedicating themselves exclusively to traditional household chores [49]. Behaviours linked to hegemonic masculinity norms, such as a man’s alcohol consumption and reports of multiple sexual partners, have been found to be associated with IPV in Uganda [50,51]. There is also a strong belief that women should not be treated the same way as men [49]. Violence against women is widely tolerated - analyses of the Ugandan national Demographic and Health Survey found that the majority of women (73%) and men (57%) believe that there are circumstances where wife beating is acceptable [52]. Research in the rural district of Rakai showed that women who perceive that their partners are at higher risk of HIV are more likely to experience IPV [51]. Qualitative research also suggests that women still fear getting tested for HIV, disclosing HIV results, and requesting condom use because of fear of IPV [50,53].

### Evaluation design

The study employs a matched cluster randomised design, with randomisation carried out within matched community pairs. As described previously, the comparison groups comprise four intervention communities (receiving the full intervention) and four control communities (receiving only Division level elements of the intervention) (Figure 3). Quantitative data collection will occur before (baseline) and four years after (follow-up) implementation of the intervention. It is important to note that a four-year follow-up period does not equate to four years of programming - barriers to programme activity including political disturbances and the suspension of activities during political election campaigns, mean that the duration of SASA! programming will have been approximately 2.8 years by the time of the follow-up survey. At each time-point, data will be collected from a cross-sectional sample of community members targeted by the intervention, and from community activists.

The primary analysis will compare intervention and control communities at follow-up. This protocol focuses on the CRT element of the evaluation that will assess the impact of the intervention on the outcomes identified in the first trial objective. However, in addition to this, monitoring and evaluation data will be collected throughout the duration of the study, including through repeated rapid assessment surveys of community members. These data will be used to document, among other things, how the intervention is implemented, its evolution in different communities, CA retention/turnover, the people it reaches, diffusion of activities into control communities, barriers to intervention delivery, and other relevant activities and policy changes that occur over the time period of the study.

A complementary ongoing programme of qualitative data collection will also allow more in-depth understanding of the implementation and processes of the intervention, including community perceptions of it, the forms of behaviour change being promoted, and the ways in which change is occurring at different levels and among different groups.

### Site selection and randomisation

Kampala comprises five administrative Divisions. While some SASA! components occur at Division-level, the majority of intervention activities are implemented in more circumscribed communities or sites. Two neighbouring Divisions were purposively chosen for implementation of the Division-level components of SASA!. Within these, eligible sites were identified on the basis of operational and programmatic considerations. Factors considered when delineating eligible sites included population density, geographical area, socioeconomic characteristics, and accessibility to the CEDOVIP office. Eight eligible sites, each comprising one or two parishes, were identified for the study. All were separated from each other by a geographical buffer (at least one parish wide) to reduce the potential for contamination between intervention and control communities.

Sites were matched into four pairs on the basis of qualitative assessments by CEDOVIP staff as to whether the site was densely populated urban or semi-rural, and assessments of the stability/mobility of the local population.

Within each pair one site was randomly selected to receive the complete intervention from the outset of the study, and the other was designated as a control that would receive the full intervention at the end of the study. The randomisation process was conducted in September 2007. The names of the two communities within a matched pair were written on identical pieces of paper which were then folded and put in a bag. One paper was blindly drawn from the bag. The selected name was assigned as an intervention community. Control communities have been receiving Division-level components of the intervention from the outset of the study.

Interviewers conducting the baseline survey were blinded to the allocation of the intervention. However, at follow-up it will not be possible to keep interviewer teams blinded.

### Recruitment and inclusion criteria

The target population for the community survey was chosen to reflect the population most likely to have had repeated and extensive contact with intervention activities and materials, those living in close proximity to the CAs. The sampling frame therefore comprises households situated in all enumeration areas (EAs) in which CAs (in intervention sites) or passive volunteers (in control sites) live.

**Figure 5:**
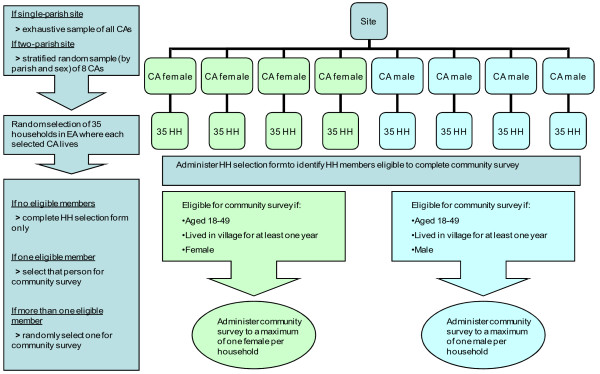
Baseline sampling strategy.

A multistage stratified random sampling process is used to sample individuals within sites (Figure 5). The primary sampling unit (PSU) is the CA/EA. The second stage of sampling comprises a simple random sample of households within each of the selected PSUs. Finally, in order to ensure the safety and confidentiality of the respondents, a maximum of one person per household is selected to complete the survey. A household selection form is used to ascertain whether the selected household has any members eligible to complete the community survey. Where more than one eligible household member is identified, one is randomly chosen for interview (with no substitutions made for refusals or failure to subsequently contact this person).

A person is eligible for inclusion if he/she is between the ages of 18 and 49 years, has lived in the village for at least a year, usually shares meals with the household, and is the same sex as the CA around whom he/she is sampled.

Separate sampling by sex was chosen both for reasons of safety (to reduce the chance that men in the immediate locality are aware of the nature of the questions in the survey and the potential disclosures that may occur), and to reflect the tendency for CAs to work more with community members of the same sex, although this is in no way circumscribed or absolute. It also makes it logistically easier to ensure that the interviewer is the same sex as the respondent.

At the time of writing, increased funding has been made available for the follow-up survey and this will thus take place in all EAs in which intervention or control CAs were recruited at the start of the study. No substitutions will be made in cases where CAs have since moved away, been substituted or been lost for other reasons. At follow-up, a new mapping exercise of households in all the EAs will be undertaken, and the same subsequent sampling stages followed as those used at baseline.

In addition to the community member’s survey, CAs and passive volunteers (in the control populations) are also surveyed at baseline and follow-up.

### Outcomes

Outcomes will be measured four years after implementation through use of an interviewer administered survey. As described previously, due to external factors forcing intermittent interruptions in programme activity, this four year follow-up period equates to approximately 2.8 years of SASA! programming.

The primary outcomes for this study are presented below, along with the hypothesised direction of intervention effect for each:

Acceptability of intimate partner violence (among all women; all men) (decrease);

Acceptability that a woman can refuse to have sex (among all women; all men) (increase);

Past year experience of physical violence from a partner (among women who have had an intimate partner in the past year) (decrease);

Past year experience of sexual violence from a partner (among women who have had an intimate partner in the past year) (decrease);

Appropriate community response to women experiencing physical and/or sexual IPV in past year (among women who experienced physical and/or sexual IPV in past year) (increase);

Past year concurrent sexual partners (among non-polygamous partnered men) (decrease).

Further discussion on the measurement of outcomes is detailed below.

#### Acceptability of violence

Questions on the acceptability of violence were adapted from those used in the WHO multi-country study on women’s health and domestic violence [54]. The original questions used in the baseline questionnaire demonstrated limited validity and reliability in this setting, and were thus adapted and added to. Focus groups and cognitive interviews were carried out to explore cultural frameworks, cognitive structures and meanings of gender-related concepts in the study communities, and a new set of questions developed and tested. Respondents will be asked a series of questions with the introduction: ‘In your opinion, does a man have good reason to hit his wife if…?’ and then presented with a range of scenarios and circumstances to which they have to provide a yes/no response (she disobeys him; she answers back to him; she disrespects his relatives; he suspects that she is unfaithful; he finds out that she has been unfaithful; she spends time gossiping with neighbours; she neglects taking care of the children; she does not complete her household work to his satisfaction; she refuses to have sexual relations with him; she accuses him of infidelity; she tells his secrets to others in the community; he is angry with her). Respondents who answer yes to at least one of these scenarios will be coded as having attitudes accepting of IPV.

#### Acceptability of a woman refusing sex

Respondents are asked the following question: ‘In your opinion, is it acceptable if a married woman refuses to have sex with her husband if she doesn’t feel like it?’ A positive response is taken to indicate acceptability of a woman refusing sex.

#### Measures of IPV

**Figure 6:**
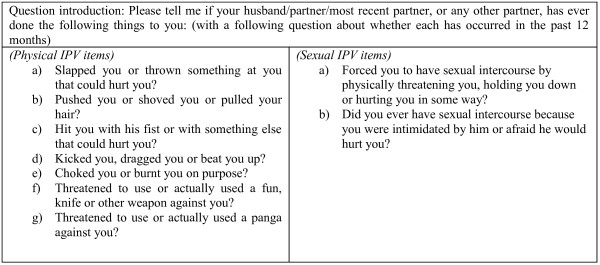
Items used to create composite outcomes for physical and sexual intimate partner violence.

Questions on the occurrence of IPV are based on questions used in the Uganda Demographic and Health Survey [42] and WHO multi-country study on women’s health and domestic violence [55]. Respondents are asked about their experiences of specific acts without reference to leading terms such as *abuse* or *violence*. Questions are shown in Figure 6. Each of the questions is asked in relation to ‘ever’ and ‘in the last 12 months’. An affirmative answer to any of the physical items for the last 12 months is taken to indicate past year physical violence; a positive answer to any of the sexual items for the last 12 months is taken to indicate past year sexual violence.

Female reports of experience are chosen over male reports of perpetration because it is anticipated that men are more likely to give socially desirable responses about their own behaviour and thus under-report perpetration. Indeed baseline data from this study revealed that male reports of perpetration were considerably lower than female reports of experience, particularly for the more severe acts [47].

Questions on violence are asked in accordance with international guidelines for the collection of data on violence against women [56]. These guidelines seek to minimise reporting bias and risk of harm posed to the respondents and interviewers involved in the survey. Interviewers, all from the local community, will undergo at least 3 weeks of preparatory training on ethical and methodological issues surrounding the conduct of a survey relating to IPV and HIV. This includes sessions on how to ensure the privacy, confidentiality and safety of the respondent, how to build rapport, and how to talk about difficult topics in a non-judgmental, sensitive and supportive manner. Interviews are conducted in Luganda or English.

#### Appropriate community response to women disclosing violence

This outcome will be measured among women reporting past year experience of physical and/or sexual IPV. Respondents will be asked: ‘When the experiences you have told me about were happening or afterwards, did anyone in your community try to help you?’ (yes/no). Those answering in the affirmative will then be read a series of questions on how that person/those people tried to help them. If the respondent reports that someone tried to help them, and they did so with at least one appropriate response, this will be coded as an appropriate community response. If the respondent reports that no one tried to help them or no one took any appropriate action or offered appropriate advice, this will be coded as a negative response. Appropriate responses reflect actions encouraged by the intervention and include someone taking the following actions: gathering other people from the community to help; knocking on the door to stop the fighting; separating the woman and her partner during fighting; informing the CA, ssenga, LC, police or other authority; talking to the woman afterwards and asking her if she wants them to help; or telling the woman to talk to someone else such as a family member, friend, community activist, LC, ssenga or other authority figure.

When interpreting results for this outcome, it is necessary to consider the complications in inference that arise when measuring intervention impact on a positive outcome that is contingent on a negative outcome (IPV) occurring. If the intervention is successful in reducing violence, then the mix of cases of violence which remain in intervention communities (and thus for which we can measure community response) may not be comparable to the mix of cases of violence occurring in control sites. They may, for example, comprise those cases that are the most hidden, and therefore least likely to elicit a community response (this in itself being a potential reason why the violence has persisted). We therefore surmise that our estimates of intervention impact for this outcome will be conservative, and we can assume a greater effect than that which we are able to observe.

#### Concurrent sexual partners

Concurrency among men partnered in the last 12 months is assessed using the following question: ‘Have you had a sexual relationship with any other woman in the last 12 months, while being with your wife/partner/most recent partner?’ Polygamous men will be excluded from the denominator as the intervention is not expected to impact on polygamous marriages over this relatively short follow-up period.

Questions on sexual behaviour are asked in accordance with widely accepted guidelines [57]. Confidentiality is stressed throughout the interview process, and as already stated interviews are conducted in private by a trained interviewer of the same sex as the respondent.

### Quantitative data collection and management

Survey tools will be used to collect data on outcome measures and on potential confounders, pathway variables and contextual variables. All completed questionnaires will be checked by field supervisors upon completion, and where problems are identified with a questionnaire it will be returned to the interviewer for corrections or for information to be completed. Once a supervisor has checked a questionnaire and cleared it as complete and satisfactory, it is sent to the Raising Voices offices. There it will be re-checked by a fieldwork coordinator or data manager. Data will be double-entered and stored in purpose built Microsoft Access databases. Where discrepancies are noted between twin-entries, reference to the original questionnaire will be made to determine the correct entry. Additional rigorous data cleaning procedures will be applied which will also entail reference to source data. Data backups will be made weekly and stored, password-protected, on a secure central drive at Raising Voices. All electronic data are anonymous. Questionnaires will be stored in locked filing cabinets in a locked office.

### Sample size

Sample size for a community randomised trial must be defined at two main levels, the level of randomisation (number of communities randomised) and the level of data collection (number of individuals surveyed in each community). In this study, a decision also had to be reached as to how many CAs to sample around within each site. For the SASA! study, sample sizes at each of these levels were decided upon based on a combination of pragmatic and theoretical considerations. The aim was to conduct the highest powered study deemed feasible given resource and staffing constraints surrounding intervention implementation and data collection. Based on these considerations, at baseline we decided on a target sample size of 200 people per site (1600 in total) for the community survey. Broken down to the different sampling levels, we aimed to complete surveys around 8 CAs in each site, and with 25 eligible people around each CA. The sample was designed to include an equal number of male and female respondents in each site.

Allowing for failure to contact respondents, refusals to participate, and households without any eligible members, we over-sampled by 40%. This figure was based on estimates of the age- and sex-distribution of Kampala’s population derived from the 2006 Uganda DHS, and male and female response rates for the same survey. It was then revised upwards to allow for the fact that recent arrivals to the parish would be ineligible to participate. Therefore, the final sample size for households to be visited was 2,240 in total (35 households around each CA).

With a final sample size of four communities and eight hundred respondents per arm (100 men and 100 women per site), we calculated the precision with which we would be able to estimate a range of effect sizes for the outcomes hypothesised to be most distal to the intervention and hardest to change, therefore, the ones for which the magnitude of change was expected to be smallest (physical and sexual IPV; and concurrent partnerships). Precision estimates, in the form of a 95% CI (the range of likely values for the true effect), are presented in Table 1. In a community randomised trial, the precision of an estimate for a given outcome and effect size is based on the number of study communities, the number of individuals surveyed in each community, and the size of the coefficient of variation (k) for the outcome of interest [58]. There are few reported data on coefficients of variation, and we therefore present precision estimates for each outcome assuming a range of different values of k. Based on other studies, we expected the value of k to be highest for past year experience of IPV (approximately 0.4) [25] which has been shown to cluster by community.

**Table 1:** Precision estimates for effect sizes given varied assumptions about control-arm prevalence and between-community variation

**Outcome**	**Estimated final sample size per site (with 4 intervention and 4 control sites in total)**	**Estimated prevalence of outcome measure**		**Effect estimate**	Estimate of precision of percentage risk difference (95% CI)*			
		**Control arm (%)**	**Intervention arm (%)**	**Risk difference (%)**	**k = 0.1**	**k = 0.2**	**k = 0.3**	**k = 0.4**
Past year experience of physical IPV (women partnered in past year)	75 (based on 100 respondents per site, 80% having partner in past year and 5% non-response to violence questions)	30.0	22.5	7.5	−2.4, 17.4^a^	−5.2, 20.2 ^a^	−8.8, 23.8 ^a^	−12.9, 27.9 ^a^
		30.0	15.0	15.0	5.8, 24.2	3.4, 26.6	0.2, 29.8	−3.4, 33.4 ^a^
		20.0	15.0	5.0	−3.2, 13.2 ^a^	−4.7, 14.7 ^a^	−6.9, 16. 9 ^a^	−9.4, 19.4 ^a^
		20.0	10.0	10.0	2.4, 17.6	1.1, 18.9	−0.8, 20.8 ^a^	−3.0, 23.0 ^a^
Past year experience of sexual IPV (women partnered in past year)	75 (based on 100 respondents per site, 80% having partner in past year and 5% non-response to violence questions)	15.0	11.0	4.0	−3.1, 11.1 ^a^	−4.1, 12.1 ^a^	−5.6, 13.6 ^a^	−7.3, 15.3 ^a^
		15.0	7.5	7.5	0.9, 14.1	0.0, 15.0	−1.3, 16.3 ^a^	−2.8, 17.8 ^a^
		10.0	7.5	2.5	−3.4, 8.4 ^a^	−3.9, 8.9 ^a^	−4.8, 9.8 ^a^	−5.8, 10.8 ^a^
		10.0	5.0	5.0	−0.4, 10.4 ^a^	−0.9, 10.9 ^a^	−1.7, 11.7 ^a^	−2.6, 12.6 ^a^
Concurrent sexual partners (among non-polygamous men partnered in the past year)	75 (based on 100 respondents per site, 80% having partner in past year and 5% non-response to sexual behaviour questions)	50.0	37.5	12.5	0.0, 25.0	−5.7, 30.7 ^a^	−12.5, 37.5 ^a^	−19.7, 44.7 ^a^
		50.0	25.0	25.0	13.4, 36.6	8.4, 41.6	2.4, 47.6	−3.9, 53.9 ^a^
		40.0	30.0	10.0	−1.3, 21.3 ^a^	−5.5, 25.5 ^a^	−10.7, 30.7 ^a^	−16.3, 36.3 ^a^
		40.0	20.0	20.0	9.5, 30.5	5.9, 34.1	1.3, 38.7	−3.7, 43.7 ^a^

*Based on calculations provided by Hayes and Bennet (1999) [58].

^a^No statistically significant difference between intervention and control groups.

*IPV*, intimate partner violence.

Using baseline data from the SASA! study, we now have a better indication of the effect sizes we will be able to detect. Baseline prevalence of past year physical IPV was 23%. Variation between communities was lower than would be expected by chance assuming a binomial model, but to allow for random error in our estimate of the coefficient of variation within matched pairs (k_m_), we conservatively assume a k_m_ value of 0.1. We should thus be able to detect a 39% or greater reduction in relative risk of past year physical IPV. Data on baseline prevalence and k_m_ for the other health related outcomes show that we should be able to detect a 58% or greater reduction in relative risk of past year sexual IPV, and a 33% or greater reduction in relative risk of past year concurrent partnering among men (Table 2).

**Table 2:** Minimum detectable risk differences for outcomes based on baseline prevalence and coefficients of variation

**Outcome**	**Baseline prevalence**	**Average number of individuals per cluster with outcome data**	**K**_**m**_	**Minimum detectable risk difference**	**Minimum detectable reduction in relative risk**
Past year physical violence (women)	23%	72	<0.1	9%	39%
Past year sexual violence (women)	12%	72	0.16	7%	58%
Past year concurrent partners (men)	45%	83	0.17	15%	33%

At the time of writing the budget for the follow-up study had increased and we have decided to increase our sample size within sites. This will give us more power to perform secondary sub-group analyses which will allow us to better understand variations in intervention impact between different sub-groups in the communities, thereby adding to our understanding of the transferability of study results. The follow-up survey will thus take place around all 96 CAs, with a target sample size of 25 eligible people around each CA and total target sample size of 2,400 individuals. With oversampling, the total number of households to be visited is '3360' in total (35 households around each CA).

### Data analysis

#### Primary analysis

The primary analysis will be a cluster-level analysis of the community survey data using a two-stage approach similar to that used in several recent studies evaluating community-based HIV and violence prevention interventions in Africa [25,33]. We will use an unpaired analysis in order to maximise statistical power given the small number of sites randomised [59], and will follow the basic principles for the analysis of cluster randomised trials as set out by Donner and Klar [60].

The analysis will be done on an intention to treat (ITT) basis, whereby data on all respondents are included whether or not they report any contact with the intervention.

Site-level past year prevalence of the outcome will be generated. The geometric mean of site-level prevalences will be calculated for intervention and control villages respectively, and the ratio of these two figures (geometric mean prevalence ratio) used to give us an estimate of crude intervention effect. An unpaired *t*-test will be used to compare the logarithms of the prevalence figures and assess the statistical significance of the difference in outcomes between intervention and control sites.

The generation of adjusted prevalence ratios for each of the outcome measures involves two stages. First an individual-level binary logistic regression model, in which the dependent variable is the binary outcome of interest, will be fitted to data from control villages. Independent variables will include measures of potential confounders (age, sex and baseline EA-level prevalence of the outcome measure of interest). This model will be used to predict the number of people in each site (intervention and control sites) that would be expected to experience the outcome at follow-up in the absence of the intervention. For each site, the ratio of observed to expected (O/E) numbers with the outcome will then be calculated. A geometric mean of these site-level summary measures will be calculated for intervention and control sites respectively, and a ratio of these means used to generate a point estimate of the adjusted intervention effect. As with the crude estimates, an unpaired t-test will be used to assess the statistical significance of this comparison and construct a 95% CI around the adjusted prevalence ratio.

#### Secondary analysis

ITT analyses demonstrate the effectiveness of offering an intervention, and the results are thus influenced by the fact that not everyone ‘complies’ with the intervention arm they are allocated to. As with all community-based interventions where the target population is the entire community, there will be some people in the SASA! communities who are not reached by intervention activities, either because of impediments to, or failures of intervention delivery at the EA level or because of personal attributes that have limited their exposure to activities that were taking place. Similarly, there will be some individuals in control communities who have received exposure to SASA!, either because of the diffusion of activities into their areas, or because of personal contact with intervention sites (contamination). We will make an assessment of individual exposure among survey respondents based on their responses to survey questions on the number of times they have seen a given set of SASA! materials, the number of times they have attended different types of activities, and the number of times they have actively participated in given types of SASA! activism.

We plan to perform a secondary analysis to assess the effect of the intervention among those in SASA! communities who report actual exposure to the intervention. Our choice of secondary analysis method reflects a supposition that lack of exposure among individuals in intervention sites will be higher than the contamination of individuals in control sites (since strong efforts have been made to minimise contamination in the context of this study).

**Figure 7:**
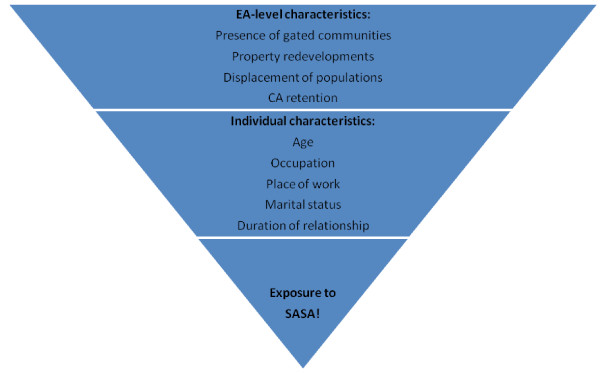
Examples of factors affecting an individual’s propensity for exposure to SASA!.

The secondary analysis will be similar to that outlined above in that it will be performed at the cluster-level and thus stick to the original randomisation employed in the allocation of the intervention. This analysis will, however involve some extra steps. Using data from intervention sites, individuals’ propensity scores for intervention exposure will be generated by entering both EA- and individual-level predictor variables (see Figure 7 for examples) into a logistic regression model where SASA! exposure is the dependent variable. Propensity scores will then be derived for individuals from control sites based on their values for the same variables. Exposed individuals from intervention sites will then be matched based on propensity score (individually matched if the pool of candidates is big enough and group matched if not) to individuals in control sites. Data from individuals in intervention sites who were not exposed to SASA!, and individuals from control sites not matched to intervention receivers, will then be excluded from the analysis.

Site-level past year prevalence of the outcome will be generated for intervention sites including only intervention recipients and control sites including only those individuals matched on propensity score to intervention recipients in intervention sites. The site-level prevalence will then be analysed in the same way as in the primary analysis, to produce both crude and adjusted estimates of intervention effect.

This analysis, restricted to those actually receiving the intervention, deals with the dilution of effects that would be observed in an ITT analysis due to incomplete exposure among individuals in intervention sites. It does not however, deal with the dilution of effects that would be caused by contamination among individuals in control sites. Indeed by matching individuals from control sites to exposed individuals in intervention sites, based on propensity for exposure, we make it more likely that we include *contaminated* individuals among our matched controls. This is not a big problem if levels of contamination are low, as anticipated. However, it becomes more of a problem, the more contamination that occurs, and thus, in such an instance an additional analysis will be required.

#### Tertiary analysis in the event of evidence of contamination among control community members

If individual reports of intervention exposure reach 10% in control sites, we will perform a supplementary per-protocol analysis. This will involve analysing individuals according to whether or not they were exposed to SASA!, regardless of which site they live in and which arm of the trial the site was allocated to. Logistic regression models will be used to assess the association between intervention exposure and each of the outcomes (intervention exposure and outcome both measured at the individual level). Multi-level adjusted models will include potential confounders, including predictors of intervention exposure, which are also hypothesised to be related to the outcome under study. Confounders will include EA-level and individual-level factors.

This analysis simultaneously overcomes dilution of effects caused by lack of universal exposure to SASA! in intervention sites, and that caused by contamination in control sites. The approach, however, is less rigorous than those outlined above as it does not stick to the unit of randomisation. Selection bias is introduced as many factors (both measured and unmeasured), which influence an individuals’ likelihood of exposure to SASA! (self-selection into the intervention group), are also directly related to their risk of experiencing the outcomes under study. While known confounders are adjusted for, there will be many such factors that are unknown and unmeasured and thus, not controlled for. Results will also be biased towards the null if non-differential misclassification of exposure occurs, something that is not unlikely with an intervention such as SASA!, where routes and nature of exposure are so diverse and complex to measure.

#### Coherence and plausibility versus statistical significance

As already highlighted, CRTs in this field of research are often restricted to a very small number of clusters, thus limiting their power to detect statistically significant results. This study is no exception. In addition to this, we know that behavioural change linked to entrenched attitudes and norms is difficult to achieve within relatively short follow-up periods. For this reason, it is possible that the magnitude of change for the IPV and concurrent partners’ outcomes might not be sufficient within this time-frame for us to achieve statistically significant results. When interpreting the results, the emphasis will therefore be on assessing whether change has occurred in the hypothesised direction and if so the magnitude of the observed effect. In particular, if the changes observed across all outcomes are in the expected direction and largely coherent with one another, this will support a plausible case for intervention impact on the intended outcomes.

#### Further analysis plans

The above analysis plans will be used for the analysis of intervention effects on the main outcome measures. Further data analysis will be completed as deemed appropriate, specifically relating to the intervention context, and mechanisms and mediators of intervention effect. Additional outcomes including behaviours and attitudes relating to broader gender norms will be explored among community members. Analysis will also be performed on data relating to the CAs, the core group expected to be most influenced by the intervention, to explore the effect the intervention has had on them as individuals, and also to assess their roles as intermediaries between the intervention and the wider community.

### Complementary programme of research

The SASA! study uses a mixed-methods approach to address a range of questions pertinent to a comprehensive evaluation of a complex social intervention such as SASA!. Complementary programmes of research taking place alongside the CRT are described in brief below. However, more detail of these components is beyond the remit of this protocol and will be presented in subsequent publications.

#### Qualitative research

Qualitative research will be conducted throughout the duration of the SASA! trial. A variety of qualitative methods (including participant observation, in-depth interviews, and focus group discussions) will be used to explore the perceptions and experiences of community members, CAs and other groups (for example, local leaders, *ssengas*, health workers) in both intervention and control sites. One strand of the qualitative research is longitudinal, comprising repeated interviews with the CAs throughout the study. A further nested, qualitative study will be performed with community members sampled on the basis of their responses to the quantitative follow-up survey. Our main areas of interest are: exploring attitudes and norms around violence, gender roles and relationship power; exploring the process of change and how people describe experiences of attitude and behaviour change; and exploring experiences of activism and how this affects individuals and those around them.

#### Process data collection

The implementation of SASA! activities will be monitored by Raising Voices staff using a set of qualitative and quantitative tools. The purpose of monitoring is to: 1) assess the frequency of and attendance at SASA! activities; 2) monitor the process of implementation and quality of activities; 3) monitor community readiness for change and the impact of activities; 4) monitor contamination, and 5) monitor background changes in policy and programming on violence and HIV.

Monitoring tools include standardised reporting forms completed by CAs; outcome tracking forms assessing the impact of SASA! activities on key outcomes, completed by senior Raising Voices/CEDOVIP staff on visits to a random selection of activities; activity report forms that monitor the process of implementation and include a quality assessment section completed by Raising Voices/CEDOVIP staff who are supporting CAs in the communities; monitoring forms completed by Raising Voices/CEDOVIP staff during ongoing training and support sessions for CAs and resource persons; a spreadsheet of external challenges faced in the implementation of SASA!; a series of rapid assessment surveys among community members to provide an indication of the stage of change reached in the community and inform the timing of implementation of each phase of SASA!; a spreadsheet monitoring where activities are taking place and contamination of control sites is occurring; and a timeline (updated on a monthly basis) of external events, policy changes and programming that occur either locally or nationally and may affect study outcomes in intervention or control sites.

### Ethics

The study has been granted ethical clearance by the Institutional Review Boards at the London School of Hygiene and Tropical Medicine, Makerere University, and the Uganda National Council for Science and Technology.

Approval for CEDOVIP and Raising Voices to implement the intervention and conduct the study in the selected sites has also been provided by the Uganda NGO Board, and by the Local Council V and III Chairpersons of the study Divisions. Agreement was also sought from local leaders at Parish- and Zone-level before any intervention implementation or data collection took place. Communities will receive ongoing feedback throughout the study.

Individual written informed consent is obtained for all community members participating in the community survey, with thumbprints used in place of signatures where respondents are unable to write. This informed consent process was designed in accordance with that used in other studies of domestic violence in societies with low literacy, and revised in accordance with local Institutional Review Board recommendations as to what was acceptable in the local context.

Of additional ethical concern is the sensitivity of the questions in the community survey. The disclosure of violence or HIV can cause distress for respondents and interviewers, while risk of harm may be posed by the partner of a respondent finding out the contents of the interview. To address these issues, the study will adhere to the WHO recommendations for conducting research on domestic violence [56]. Actions include the careful wording of questions to ensure that they are non-judgmental, providing interviewers with intensive training on how to respond if someone discloses violence or requests assistance, providing all participants with information about potential sources of support, and ensuring that follow-up support can be made available if requested. The questionnaire also includes scripted endings for women who disclose violence, which aim to provide basic messages about the respondent’s strengths and the unacceptability of violence.

## Discussion

The SASA! study is one of few cluster randomised trials to assess the impact of a gender focused structural intervention on IPV and HIV prevention. It uses a rigorous methodology to minimise several forms of selection and measurement bias that have to date impeded interpretation of many evaluations of complex community-based interventions.

Selection bias is kept to a minimum in a variety of ways. The cluster randomised design eliminates placement bias with regards to where the intervention is implemented, and the matched design attempts to ensure that intervention and comparison communities are similar despite the low number of communities randomised. Within sites, sampling of households occurs in pre-specified areas around community volunteers. The standardised process of volunteer recruitment across both intervention and control sites reduces systematic bias in the intervention/control comparison that may otherwise occur if those who volunteer tend to live in particular types of areas not representative of the sites as a whole. Furthermore, the survey is conducted in a random sample of households in these pre-specified areas, and an intention to treat analysis will measure the overall community impact of the intervention rather than intervention effects among individuals choosing to participate in activities. In this way it is not affected by self-selection bias that may be introduced where inclusion in the study depends on intervention uptake (and thus perhaps underlying personal empowerment).

Selection bias can also occur through non-response, potentially affecting the generalisability of study results as well as the internal validity of the intervention/control comparison. Response rates are maximised in the SASA! study in a number of ways: at least three repeat visits are made to households where respondents are not available at the time of the first visit; interviewers are trained on how to introduce the study and build rapport with respondents in order to minimise the number of refusals; the cross-sectional design precludes the problem of loss to follow-up between survey rounds.

Measurement bias is minimised through the use of standardised questions with demonstrated validity and reliability that are administered by interviewers who have undergone three weeks intensive training on conducting surveys related to IPV and sexual behaviour.

Despite these strengths, the study also has several limitations. A number of factors might lead us to underestimate true intervention effects. Firstly, contamination may occur between intervention and control sites, caused by diffusion of intervention activities, and the movement of people between sites. While we have ensured geographical buffers between sites, the overall study area is small and the high levels of population mobility observed in these communities, in combination with the fact that social diffusion is at the heart of the SASA! intervention, mean that some contamination is likely.

On a related theme, some basic elements of the intervention are implemented at Division-level. Because these elements cannot be implemented lower down (for example, work with hospitals that serve the whole Division), and because it was not logistically feasible to randomise whole Divisions, both intervention and control sites are concentrated in two Divisions that receive the Division-level components of the intervention. The presence of this low-intensive intervention in control sites might weaken estimates of the intervention effect. Effect estimates should be interpreted as the added value of the intensive local components of the intervention when implemented alongside the Division-level components, rather than the impact of the package as a whole versus nothing.

Another factor that might cause us to underestimate intervention effects is the limited duration of follow-up in the study. Our measures of intervention impact will only capture changes that occur within four years of intervention implementation. Duration of exposure to the intervention is in effect shorter than this (approximately 2.8 years) due to intermittent interruptions to programme activity caused by political unrest and election campaigning. This is a relatively short time in which to make an impact on behavioural outcomes, especially since the intervention takes communities through the stages of change model before attempting to promote action. The SASA! study will thus be limited in its capacity to address questions of the intervention’s potential in the longer-term.

An additional complicating factor related to duration of intervention exposure is that the study is conducted in highly mobile populations. This, coupled with the cross-sectional design of the follow-up survey, means we may survey people who have not been exposed to the intervention for an extended period. Although we attempt to overcome this problem by restricting eligibility to those who have lived in their village for at least one year, if the intervention takes longer than a year to have an impact, high levels of population mobility might affect the study’s capacity to detect an impact. This limitation is not specific to the evaluation design but instead pertains to whether the intervention itself can promote community-wide change in an unstable population. The SASA! study will thus answer the question whether a community-level impact can be observed despite high levels of mobility, rather than measure the potential efficacy of the intervention given ideal experimental conditions.

The above limitations would lead us to underestimate any true potential (or adverse consequences) of the SASA! intervention, and should therefore be borne in mind when interpreting null associations, rather than invoked to undermine observed intervention effects.

The repeated cross-sectional design is arguably a further limitation. While baseline data will allow us to assess the comparability of areas at baseline (and control for differences in the analysis), the cross-sectional nature of the follow-up survey makes it harder to definitively separate intervention effects from the effects of changes in population make-up during the course of the study, especially if population mobility is high. As discussed, the most likely way that random population changes would influence effect estimates is to bias them towards unity. The repeated cross-sectional design was chosen in preference to a cohort study as the latter was deemed both impractical in this high mobility setting, and subject to selection bias from excessive levels of attrition. The cross-sectional design also suits our objective to measure community- rather than individual-level intervention impact.

Finally, as with many trials of community-based interventions, the number of communities randomised is low. Our effect estimates may therefore have relatively low precision, particularly if there is a high level of clustering of outcomes or effect sizes are small. The rigorous design will, however, produce unbiased estimates of impact, and we will thus focus on the consistency, congruency and plausibility of results rather than the statistical significance of individual effect estimates.

Although subject to several limitations, the SASA! study is one of a small number of cluster randomised trials to assess the impact of this type of community-based intervention on IPV and sexual behaviour. The intervention consists of a combination of complementary components, implemented by a wide range of community actors. In contrast to many other health promotion approaches, the focus of the intervention is to support a process of change at a community level, with the aim of sensitising, engaging with and mobilising communities to address gender norms, violence and HIV risk behaviours in their communities. For this reason, the specifics of some of the intervention activities are not proscribed on a per protocol basis, but rather develop and evolve in direct response to community priorities, needs and issues. In designing the SASA! study, we faced not only the usual logistical constraints, but also the difficulties of implementing such a holistic and broad intervention within the context of a randomised trial. We chose to restrict the trial to a small number of sites, placing emphasis on intervention quality and adherence to the trial protocol. The CRT will be complemented by an ongoing process evaluation and programme of qualitative research. This will allow us to address questions not only of intervention impact and mechanisms of action, but also its feasibility, acceptability, potential transferability to other contexts and cost.

Despite widespread commitments to addressing gender based violence and HIV, this trial is one of a very limited number of CRTs that have been conducted in sub-Saharan Africa on this issue. Given the complexity and challenges involved in conducting this form of research, the trial findings will not only provide important evidence to help inform future policy, but will also provide an important opportunity to learn more about the feasibility of this approach to evaluating interventions that are focused on social determinants of IPV and HIV risk.

## Trial status

At the point of submission, the follow-up survey is just underway – the expected duration of data collection is 3 months from this point.

## Abbreviations

CA: Community activist; CEDOVIP: Centre for Domestic Violence Prevention, Kampala; CRT: Cluster randomised trial; DHS: Demographic and Health Survey; EA: Enumeration area; IPV: Intimate partner violence; ITT: Intention to treat (analysis); M&E: Monitoring and evaluation; PSU: Primary sampling unit; UNAIDS: Joint United Nations Programme on HIV/AIDS; WHO: World Health Organisation.

## Competing interests

Lori Michau is co-Director of Raising Voices and designed the SASA intervention. Janet Nakuti is the Monitoring and Evaluation Officer for Raising Voices. Tina Musuya is the Director of CEDOVIP and in charge of the implementation of the SASA! intervention being evaluated. They have played a central role in ensuring the appropriate conceptualisation and implementation of the evaluation, including the topics covered in the study questionnaire, the implementation of the fieldwork, and ensuring the provision of support to women requesting assistance. They have had no involvement in the randomisation of matched community pairs, no direct involvement in data collection for the CRT, and will have no involvement with the data analysis.

## Authors’ contributions

CW, LM and TM are Principal Investigators on the study, responsible for the overall conceptualisation, design and management of the study. TA, KD and LK are social epidemiologists responsible for major aspects of study design, sampling, development of research instruments, interviewer training and supervision of the quantitative survey. LF coordinated the development of the baseline questionnaire and coordinated and supervised baseline data collection. JN has input to the development and implementation of monitoring and evaluation instruments, the development of the follow-up questionnaire, and interviewer training, as well as being involved in baseline data collection and data management. NK is the social scientist leading the qualitative research and analysis, and supporting the implementation of the follow-up survey. ES had major input to the development and implementation of the follow-up survey. DK supported several aspects of the research, in particular supporting the implementation of fieldwork procedures. TA led the development of the study protocol and produced the first draft of this manuscript. All authors have commented on and offered edits to the original draft, and all have read and approved the final version.
